# Identification of *Cytospora* (*Cytosporaceae*, *Diaporthales*) species associated with poplar and willow canker diseases in Xizang, China

**DOI:** 10.3897/mycokeys.132.187445

**Published:** 2026-05-08

**Authors:** Jiangrong Li, Yi Li, Yonglin Wang, Ning Jiang

**Affiliations:** 1 Key Laboratory of Forest Ecology in Tibet Plateau, Ministry of Education, Institute of Tibet Plateau Ecology, Xizang Agricultural and Animal Husbandry University, Linzhi, Xizang 860000, China Chinese Academy of Forestry Beijing China; 2 The Key Laboratory for Silviculture and Conservation of the Ministry of Education, Beijing Forestry University, Beijing 100083, China Beijing Forestry University Beijing China; 3 Key Laboratory of Forest Protection of National Forestry and Grassland Administration, Ecology and Nature Conservation Institute, Chinese Academy of Forestry, Beijing 100091, China Xizang Agricultural and Animal Husbandry University Linzhi China

**Keywords:** *
Ascomycota
*, *
Diaporthales
*, multi-locus phylogeny, *
Salicaceae
*, systematics

## Abstract

*
Cytospora* is a species-rich and globally distributed genus in *Diaporthales*, characterized by hyaline, aseptate, and allantoid ascospores and conidia. Many species in this genus are recognized as important plant pathogens that cause branch cankers on a wide range of hosts. Recent taxonomic revisions, integrating morphological and molecular phylogenetic data, have clarified the generic limits of *Cytospora*, resulting in the recognition of three groups and eight types in the teleomorph and three groups and thirteen types in the anamorph, as well as ten species complexes. In this study, canker diseases on *Populus szechuanica* var. *tibetica* and *Salix lasiogyne* were investigated in Xizang, China. Species identification was conducted using a polyphasic approach combining morphological characteristics and multi-locus phylogenetic analyses based on ITS, *act*, *rpb*2, *tef*1, and *tub*2 sequences. Consequently, two new species, *Cytospora chayuensis* from the *C. leucostoma* species complex and *C. sinopopuli* from the *C. kantschavelii* species complex, are introduced herein. This study contributes to knowledge of *Cytospora* species diversity and provides foundational data for the management of poplar and willow canker diseases.

## Introduction

Forest ecosystems are indispensable for maintaining global ecological balance but are increasingly threatened by fungal diseases, particularly cankers and dieback caused by members of *Ascomycota* (Adams et al. 2006; Fan et al. 2015a; Lawrence et al. 2017; Jiang et al. 2023, 2024, 2025b, 2025c; Li et al. 2024a). The plant family *Salicaceae*, specifically the genera *Populus* and *Salix*, comprises economically and ecologically important tree species widely used in agroforestry and shelterbelt construction. However, these trees are highly susceptible to canker diseases, which result in substantial economic losses and ecological degradation (Fan et al. 2015b, 2020; Wang et al. 2015; Lawrence et al. 2018; Azizi et al. 2024; Lin et al. 2024). Among the diverse assemblage of causal agents, the genus *Cytospora* (*Cytosporaceae*, *Diaporthales*) is one of the most widespread and destructive groups (Norphanphoun et al. 2017; Lin et al. 2022, 2023; Ilyukhin et al. 2023; Wang et al. 2024; Jiang et al. 2025a). Exhibiting a versatile lifestyle, *Cytospora* species frequently exist as latent endophytes in healthy tissues but can transition into an aggressive pathogenic phase when hosts are subjected to environmental stresses such as drought, frost, or nutrient deficiency (Sessa et al. 2018; Dudley et al. 2020). This dual lifestyle has complicated efforts to understand its taxonomy.

The taxonomy of *Cytospora* has historically been one of the most complex chapters in mycology. Since the genus was established by Ehrenberg in 1818, its classification has undergone substantial revisions (Ehrenberg 1818; Adams et al. 2002, 2006; Fan et al. 2020; Lin et al. 2024; Jiang et al. 2025a). For nearly two centuries, the taxonomy was governed by a dual nomenclature system in which asexual morphs were classified as *Cytospora*, whereas their sexual counterparts were placed in genera such as *Valsa*, *Leucostoma*, *Valsella*, and *Valseutypella* based on stromatal characteristics (Wehmeyer 1975; Spielman 1985). In addition, species identification traditionally relied heavily on host association under the assumption of strict host specificity (Ehrenberg 1818; Wehmeyer 1975; Adams et al. 2002). The reliance on variable morphological traits, such as stromatal size or locule configuration, which may fluctuate depending on substrate and culture conditions, further obscured true species boundaries (Fan et al. 2020; Lin et al. 2024; Jiang et al. 2025a).

The introduction of molecular phylogenetics and the implementation of the “One Fungus, One Name” principle have fundamentally reshaped the systematics of this group (Taylor 2011; Rossman 2014). Phylogenetic studies have confirmed that *Cytosporaceae* comprises a single genus, *Cytospora*, which now encompasses species previously assigned to *Valsa*, *Leucostoma*, *Valsella*, *Valseutypella*, and *Leucocytospora* (Rossman et al. 2015). Modern species delimitation in *Cytospora* therefore relies on a polyphasic approach, integrating morphological characterization with multi-locus phylogenetic analyses (Adams et al. 2002; Fan et al. 2020). The internal transcribed spacer (ITS) region alone often provides insufficient resolution for distinguishing closely related species; consequently, additional loci such as actin (*act*), RNA polymerase II subunit 2 (*rpb*2), translation elongation factor 1-alpha (*tef*1), and beta-tubulin (*tub*2) are widely used as secondary DNA barcodes (Pan et al. 2021; Hanifeh et al. 2022; Lin et al. 2024).

Recent comprehensive taxonomic revisions have further clarified the phylogenetic framework of *Cytospora*. Current classifications recognize ten major species complexes, including the *Cytospora chrysosperma* species complex, *Cytospora leucostoma* species complex, and *Cytospora kantschavelii* species complex, together with several singleton species (Lin et al. 2024; Jiang et al. 2025a). Morphologically, species are categorized into distinct morphotypes consisting of three groups (SI–SIII) with eight types in the teleomorph and three groups (AI–AIII) with thirteen types in the anamorph (Lin et al. 2024). Interestingly, some lineages show strong host associations; for example, species within the *Cytospora chrysosperma* species complex are frequently associated with hosts in the *Salicaceae* (Lin et al. 2024; Jiang et al. 2025a), suggesting potential host-driven evolutionary patterns.

Despite these advances, the diversity of *Cytospora* species in many biodiversity hotspots remains poorly explored, particularly in high-altitude forest ecosystems (Li et al. 2024a; Jiang et al. 2025a). The Xizang Autonomous Region of China, often referred to as the “Third Pole,” is characterized by unique plateau ecosystems ranging from alpine shrublands to subalpine forests (Li et al. 2024b). This region supports diverse endemic plant communities and may harbor a rich, yet largely unexplored, fungal diversity (Jiang et al. 2025a). *Populus* and *Salix* are important pioneer tree species in these plateau environments and play key roles in wind protection and ecosystem stabilization. However, the microfungal communities associated with these hosts in Xizang remain insufficiently studied.

During a recent survey of forest pathogens in Xizang, severe branch dieback and canker symptoms were observed on poplar and willow trees. To clarify the etiology of these diseases and document the fungal diversity associated with *Salicaceae* in this region, the present study employed an integrative taxonomic approach. Detailed morphological examinations were conducted according to the recently proposed classification framework, and phylogenetic analyses were performed using a five-gene dataset (ITS, *act*, *rpb*2, *tef*1, and *tub*2). The objectives of this study were to identify *Cytospora* species associated with canker diseases of *Salicaceae* in Xizang and to describe new taxa based on combined morphological and molecular evidence.

## Materials and methods

### Sample collection and isolation

Field expeditions were undertaken in the Xizang Autonomous Region, China, during 2024 to investigate forest pathogens. Fresh specimens exhibiting typical canker symptoms were collected from *Populus szechuanica* var. *tibetica* and *Salix lasiogyne*. Infected branches were cut into appropriate segments and placed in paper bags for transport to the laboratory.

Fungal isolates were established using a single-spore technique. Mucilaginous spore masses emerging from the fruiting bodies were removed using a sterile needle and suspended in sterile distilled water. The resulting suspension was spread onto the surface of potato dextrose agar (PDA, 200 g potatoes, 20 g dextrose, and 20 g agar per L) plates and incubated at 25 °C. Germinating spores were examined using a stereomicroscope after 24 h. Single germinating spores were then transferred to fresh PDA plates to obtain pure cultures. Colony morphology and growth characteristics were recorded after incubation at 25 °C in the dark. Voucher specimens were deposited in the Herbarium of the Chinese Academy of Forestry (CAF), Beijing, China, and living cultures were deposited in the China Forestry Culture Collection Center (CFCC), Beijing, China.

### Morphological analysis

Macromorphological features of the stromata on host substrates were examined and photographed using a Zeiss Discovery V8 stereomicroscope (Oberkochen, Germany). For micromorphological observations, hand-sectioned fruiting bodies were mounted in sterile distilled water and examined using an Olympus BX51 light microscope (Tokyo, Japan) equipped with differential interference contrast (DIC). Detailed characteristics of the teleomorph (ascomata, asci, and ascospores) and anamorph (conidiomata, conidiophores, conidiogenous cells, and conidia) were captured and measured.

Dimensions for spores were determined by measuring at least 50 randomly selected ascospores and conidia. The measurement data were expressed in the format: (minimum–)(mean – standard deviation) – (mean + standard deviation)(–maximum). Colony morphology, including texture, density, and color (both surface and reverse), was assessed on PDA and MEA plates after incubation at 25 °C for 14 days.

### DNA extraction, PCR amplification, and phylogenetic analysis

Total genomic DNA was isolated from fresh fungal mycelium harvested from colonies grown on PDA for 10 d, utilizing the CTAB extraction protocol. Five genomic loci were targeted for amplification and sequencing: ITS, *act*, *rpb*2, *tef*1, and *tub*2. The following primer pairs were employed: ITS1/ITS4 for ITS (White et al. 1990); ACT512F/ACT783R for *act* (Carbone and Kohn 1999); RPB2-5F/RPB2-7cR for *rpb*2 (Liu et al. 1999); EF1-728F/EF1-986R for *tef*1 (Carbone and Kohn 1999); and Bt2a/Bt2b for *tub*2 (Glass and Donaldson 1995).

PCR amplifications were performed in a 25 μL reaction volume consisting of 12.5 μL of 2× Master Mix, 1 μL of genomic DNA template, 1 μL of each forward and reverse primer (10 μM), and 9.5 μL of ddH_2_O. Polymerase chain reaction (PCR) was performed under the following conditions: initial denaturation at 94 °C for 5 min, followed by 35 cycles of denaturation at 94 °C for 30 s, annealing at 48 °C (for ITS), 54 °C (for *tef*1 and *tub*2), or 55 °C (for *act* and *rpb*2) for 50 s, and extension at 72 °C for 1 min, with a final elongation step at 72 °C for 7 min. The resulting PCR products were assessed by electrophoresis on a 1% agarose gel and subsequently sequenced bi-directionally by Ruibiotech Co., Ltd. (Beijing, China).

Raw sequence data were assembled and edited using MEGA v.7 (Kumar et al. 2016) and were compared using a megablast search of the NCBI GenBank nucleotide database to identify the most closely related *Cytospora* species for each strain. Based on Jiang et al. (2025a), CFCC 71370 and CFCC 71955 were assigned to the *Cytospora kantschavelii* species complex, while CFCC 71396 and CFCC 71397 were assigned to the *Cytospora leucostoma* species complex, allowing for accurate species identification within the species complexes.

The newly generated sequences in this study were deposited in GenBank and integrated with relevant reference sequences (Table 1). Multiple sequence alignments for individual loci were generated using MAFFT v.7.110 (Katoh and Standley 2013) and manually optimized where necessary. The five individual datasets were then concatenated into a supermatrix using SequenceMatrix v.1.7.8.

**Table 1.** GenBank accession numbers used in the phylogenetic analyses.

Species	Strain	GenBank accession numbers					Reference
		ITS	*act*	*rpb*2	*tef*1	*tub*2	
* Cytospora azerbaijanica*	IRAN 4201C^T^	MW295526	MZ014513	MW824360	MW394147	NA	Hanifeh et al. 2022
* Cytospora azerbaijanica*	IRAN 4627C	OM368650	NA	NA	OM372512	NA	Hanifeh et al. 2022
* Cytospora alba*	CFCC 55462 ^T^	MZ702593	OK303457	OK303516	OK303577	OK303644	Lin et al. 2022
* Cytospora alba*	CFCC 55463 ^T^	MZ702594	OK303458	OK303517	OK303578	OK303645	Lin et al. 2022
* Cytospora albodisca*	CFCC 53161 ^T^	MW418406	MW422899	MW422909	MW422921	MW422933	Pan et al. 2021
* Cytospora albodisca*	CFCC 54373	MW418407	MW422900	MW422910	MW422922	MW422934	Pan et al. 2021
* Cytospora albodisca*	CFCC 58440	PP988734	NA	PQ074909	PQ074270	PQ075225	Pan et al. 2021
* Cytospora amygdali*	CBS 144233 ^T^	MG971853	MG972002	NA	MG971659	NA	Lawrence et al. 2018
* Cytospora balanejica*	IRAN 4419C ^T^	MZ948960	MZ997842	MZ997845	MZ997848	NA	Azizi et al. 2024
* Cytospora beijingensis*	CFCC 55835 ^T^	PP988743	PQ074603	PQ074915	PQ074278	PQ075232	Lin et al. 2024
* Cytospora beijingensis*	CFCC 55836 ^T^	PP988744	PQ074604	PQ074916	PQ074279	PQ075233	Lin et al. 2024
* Cytospora breviconidialis*	CFCC 71020 ^T^	PQ778513	PV454724	PV461905	PV467134	PV467258	Jiang et al. 2025a
* Cytospora breviconidialis*	CFCC 71320 ^T^	PQ778514	PV454725	PV461906	PV467135	PV467259	Jiang et al. 2025a
* Cytospora brunnea*	CFCC 71082 ^T^	PQ778532	PV454738	NA	PV467153	PV467277	Jiang et al. 2025a
* Cytospora brunnea*	CFCC 71322 ^T^	PQ778533	PV454739	NA	PV467154	PV467278	Jiang et al. 2025a
* Cytospora cafii*	CFCC 71119 ^T^	PQ778515	PV454726	PV461907	PV467136	PV467260	Jiang et al. 2025a
* Cytospora caileii*	CFCC 71038 ^T^	PQ778516	PV454727	PV461908	PV467137	PV467261	Jiang et al. 2025a
* Cytospora caileii*	CFCC 71039 ^T^	PQ778517	PV454728	PV461909	PV467138	PV467262	Jiang et al. 2025a
* Cytospora caileii*	CFCC 71088 ^T^	PQ778518	PV454729	PV461910	PV467139	PV467263	Jiang et al. 2025a
* Cytospora caileii*	CFCC 71108	PQ778519	PV454730	PV461911	PV467140	PV467264	Jiang et al. 2025a
* Cytospora cerebriformis*	CFCC 50020 ^T^	MH933638	MH933545	NA	NA	NA	Pan et al. 2018
* Cytospora cerebriformis*	CFCC 50023	KR045635	KU711003	KU710964	KU710926	KR045676	Hyde et al. 2014
* Cytospora cerebriformis*	CFCC 59051	PP988769	NA	PQ074938	PQ074300	PQ075251	Lin et al. 2024
* Cytospora cerebriformis*	CFCC 59061 ^T^	PP988770	NA	PQ074939	PQ074301	PQ075252	Lin et al. 2024
* Cytospora cerebriformis*	CFCC 59104	PQ778527	NA	PV461918	PV467148	PV467272	Jiang et al. 2025a
* Cytospora cerebriformis*	CFCC 71305	PQ778528	PV454736	PV461919	PV467149	PV467273	Lin et al. 2024
* Cytospora cerebriformis*	CFCC 71418	PQ778529	PV454737	PV461920	PV467150	PV467274	Lin et al. 2024
*** Cytospora chayuensis* sp. nov**.	**CFCC 71396** ^T^	**PX974048**	**PX981928**	**PX981932**	**PX981936**	**PX981940**	**This study**
*** Cytospora chayuensis* sp. nov**.	**CFCC 71397** ^T^	**PX974049**	**PX981929**	**PX981933**	**PX981937**	**PX981941**	**This study**
* Cytospora cinnamomea*	CFCC 53178 ^T^	MK673054	MK673024	NA	NA	MK672970	Pan et al. 2020
* Cytospora corylina*	CFCC 54684 ^T^	MW839861	MW815937	MW815951	MW815886	MW883969	Gao et al. 2021
* Cytospora crassa*	CFCC 71029 ^T^	PQ778536	PV454742	PV461927	PV467157	PV467281	Jiang et al. 2025a
* Cytospora curvata*	MFLUCC 15-0865 ^T^	KY417728	KY417694	NA	NA	NA	Norphanphoun et al. 2017
* Cytospora davidiana*	CXY 1374 ^T^	KM034869	NA	NA	NA	KM034901	Wang et al. 2015
* Cytospora deqinensis*	CFCC 58467 ^T^	PP988795	PQ074646	PQ074961	PQ074325	PQ075275	Lin et al. 2024
* Cytospora deqinensis*	CFCC 58468	PP988796	PQ074647	PQ074962	PQ074326	PQ075276	Lin et al. 2024
* Cytospora deqinensis*	CFCC 71031	PQ778538	PV454744	PV461929	PV467159	PV467283	Jiang et al. 2025a
* Cytospora deqinensis*	CFCC 58192	PP988794	PQ074645	NA	PQ074324	PQ075274	Lin et al. 2024
* Cytospora deqinensis*	CFCC 58469	PP988797	PQ074648	PQ074963	PQ074327	PQ075277	Lin et al. 2024
* Cytospora deqinensis*	CFCC 58625	PP988798	PQ074649	NA	PQ074328	NA	Lin et al. 2024
* Cytospora deqinensis*	CFCC 58626 ^T^	PP988799	NA	NA	PQ074329	NA	Lin et al. 2024
* Cytospora deqinensis*	CFCC 71417	PQ778539	PV454745	PV461930	PV467160	PV467284	Jiang et al. 2025a
* Cytospora diqingensis*	CFCC 58244 ^T^	PP988804	PQ074652	PQ074966	PQ074332	PQ075280	Lin et al. 2024
* Cytospora donetzica*	MFLUCC 16-0574 ^T^	KY417731	KY417697	KY417799	NA	NA	Norphanphoun et al. 2017
* Cytospora donglingensis*	CFCC 53159 ^T^	MW418412	MW422903	MW422915	MW422927	MW422939	Pan et al. 2021
* Cytospora donglingensis*	CFCC 53160	MW418414	MW422905	MW422917	MW422929	MW422941	Pan et al. 2021
* Cytospora donglingensis*	CFCC 58206	PP988810	PQ074657	PQ074972	PQ074338	PQ075286	Lin et al. 2024
* Cytospora donglingensis*	CFCC 58207	PP988811	PQ074658	PQ074973	PQ074339	PQ075287	Lin et al. 2024
* Cytospora donglingensis*	CFCC 58215	PP988812	NA	PQ074974	PQ074340	PQ075288	Lin et al. 2024
* Cytospora donglingensis*	CFCC 58218	PP988813	NA	PQ074975	PQ074341	PQ075289	Lin et al. 2024
* Cytospora donglingensis*	CFCC 58220	PP988814	NA	PQ074976	PQ074342	PQ075290	Lin et al. 2024
* Cytospora donglingensis*	CFCC 58436	PP988815	NA	PQ074977	PQ074343	PQ075291	Lin et al. 2024
* Cytospora donglingensis*	CFCC 58491	PP988816	PQ074659	PQ074978	PQ074344	PQ075292	Lin et al. 2024
* Cytospora donglingensis*	CFCC 58494	PP988817	PQ074660	PQ074979	PQ074345	NA	Lin et al. 2024
* Cytospora donglingensis*	CFCC 71101	PQ778542	PV454748	PV461933	PV467163	PV467287	Jiang et al. 2025a
* Cytospora donglingensis*	CFCC 71416	PQ778543	PV454749	PV461934	PV467164	PV467288	Jiang et al. 2025a
* Cytospora elaeagnina*	CFCC 56017 ^T^	PP988820	PQ074662	PQ074982	PQ074348	PQ075293	Lin et al. 2024
* Cytospora elaeagnina*	CFCC 56018 ^T^	PP988821	PQ074663	PQ074983	PQ074349	PQ075294	Lin et al. 2024
* Cytospora euonymina*	CFCC 89993 ^T^	MH933630	MH933537	MH933600	MH933505	MH933590	Pan et al. 2018
* Cytospora fuckeliana*	CBS 113699 ^T^	PP988847	PQ074685	PQ075005	PQ074373	PQ075317	Lin et al. 2024
* Cytospora gansuensis*	JZB3670130 ^T^	PQ013683	NA	PQ053497	PQ044439	PQ053519	He et al. 2024
* Cytospora gigaspora*	CFCC 50014	KR045630	KU710999	KU710959	KU710922	KR045671	Fan et al. 2015c
* Cytospora gigaspora*	CFCC 89634 ^T^	KF765671	KU711000	KU710960	KU710923	KR045672	Fan et al. 2015c
* Cytospora gigaspora*	CFCC 71273	PQ778551	PV454756	PV461942	NA	PV467296	Jiang et al. 2025a
* Cytospora hoffmannii*	CBS 258.34 ^T^	PP988861	PQ074695	PQ075019	PQ074386	PQ075330	Lin et al. 2024
* Cytospora kantschavelii*	CFCC 58219	PP988876	PQ074707	PQ075029	PQ074399	PQ075341	Lin et al. 2024
* Cytospora kantschavelii*	MFLUCC 15-0857 ^T^	KY417738	KY417704	KY417806	NA	NA	Norphanphoun et al. 2017
* Cytospora kantschavelii*	CBS 485.63	PP988872	NA	NA	PQ074395	PQ075338	Lin et al. 2024
* Cytospora kantschavelii*	CFCC 58194	PP988873	PQ074704	NA	PQ074396	PQ075339	Lin et al. 2024
* Cytospora kantschavelii*	CFCC 58205	PP988874	PQ074705	PQ075027	PQ074397	NA	Lin et al. 2024
* Cytospora kantschavelii*	CFCC 58213	PP988875	PQ074706	PQ075028	PQ074398	PQ075340	Lin et al. 2024
* Cytospora kantschavelii*	CFCC 59071	PP988877	PQ074708	PQ075030	PQ074400	PQ075342	Lin et al. 2024
* Cytospora kantschavelii*	CXY 1386	KM034867	NA	NA	NA	NA	Wang et al. 2015
* Cytospora kantschavelii*	MFLUCC 16-0575	KY417739	KY417705	KY417807	NA	NA	Norphanphoun et al. 2017
* Cytospora kunsensis*	CFCC 59570 ^T^	PP060459	PP059661	PP059665	PP059671	PP059677	Wang et al. 2024
* Cytospora lauricola*	CFCC 58193 ^T^	PP988881	PQ074711	PQ075033	PQ074404	PQ075345	Lin et al. 2024
* Cytospora lauricola*	CFCC 58208	PP988882	PQ074712	NA	PQ074405	NA	Lin et al. 2024
* Cytospora lauricola*	CFCC 58221 ^T^	PP988883	PQ074713	PQ075034	PQ074406	PQ075346	Lin et al. 2024
* Cytospora leucostoma*	CBS 133.76	PP988900	PQ074730	PQ075050	PQ074423	PQ075363	Lin et al. 2024
* Cytospora leucostoma*	CFCC 50022	MH933627	MH933534	NA	MH933502	MH933569	Pan et al. 2018
* Cytospora leucostoma*	CFCC 53163	MK673059	MK673029	MK673000	MK672948	MK672975	Pan et al. 2020
* Cytospora lijiangensis*	CFCC 58209 ^T^	PP988903	PQ074733	NA	PQ074426	PQ075366	Lin et al. 2024
* Cytospora lijiangensis*	CFCC 58483 ^T^	PP988905	PQ074735	PQ075054	PQ074428	NA	Lin et al. 2024
* Cytospora lijiangensis*	CFCC 59069	PP988907	PQ074737	PQ075056	PQ074430	PQ075369	Lin et al. 2024
* Cytospora lijiangensis*	CFCC 58453	PP988904	PQ074734	PQ075053	PQ074427	PQ075367	Lin et al. 2024
* Cytospora lijiangensis*	CFCC 58488	PP988906	PQ074736	PQ075055	PQ074429	PQ075368	Lin et al. 2024
* Cytospora lijiangensis*	CFCC 59074	PP988908	PQ074738	PQ075057	PQ074431	PQ075370	Lin et al. 2024
* Cytospora lijiangensis*	CFCC 89999	MH933631	MH933538	MH933601	MH933506	MH933591	Lin et al. 2024
* Cytospora mali-spectabilis*	CFCC 53181 ^T^	MK673066	MK673036	MK673006	MK672953	MK672982	Pan et al. 2020
* Cytospora megaspora*	CFCC 71033 ^T^	PQ778566	PV454769	PV461955	PV467185	PV467307	Jiang et al. 2025a
* Cytospora megaspora*	CFCC 71040	PQ778567	PV454770	PV461956	PV467186	PV467308	Jiang et al. 2025a
* Cytospora megaspora*	CFCC 71302	PQ778568	PV454771	PV461957	PV467187	PV467309	Jiang et al. 2025a
* Cytospora multiseriata*	CFCC 58707 ^T^	PP988920	PQ074749	PQ075068	NA	PQ075382	Lin et al. 2024
* Cytospora multiseriata*	CFCC 58874	PQ778571	PV454772	PV461958	NA	PV467310	Jiang et al. 2025a
* Cytospora multiseriata*	CFCC 71117	PQ778573	PV454774	PV461960	PV467189	PV467312	Jiang et al. 2025a
* Cytospora multiseriata*	CFCC 71120	PQ778574	PV454775	PV461961	PV467190	PV467313	Jiang et al. 2025a
* Cytospora neolhasaensis*	CFCC 58706 ^T^	PP988902	PQ074732	PQ075052	PQ074425	PQ075365	Lin et al. 2024
* Cytospora nitschkeana*	CBS 118.22^T^	PP988924	PQ074752	PQ075071	PQ074445	PQ075385	Lin et al. 2024
* Cytospora nitschkeana*	CBS 195.42	PP988925	PQ074753	PQ075072	PQ074446	PQ075386	Lin et al. 2024
* Cytospora nitschkeana*	CBS 196.42	PP988926	PQ074754	PQ075073	PQ074447	PQ075387	Lin et al. 2024
* Cytospora notastroma*	NE_TFR5	JX438632	NA	NA	JX438543	NA	Kepley et al. 2015
* Cytospora olivacea*	CFCC 53176 ^T^	MK673068	MK673038	MK673008	MK672955	MK672984	Pan et al. 2020
* Cytospora paracinnamomea*	CFCC 55453 ^T^	MZ702594	OK303456	OK303515	OK303576	OK303643	Lin et al. 2024
* Cytospora paracinnamomea*	CFCC 55455 ^T^	MZ702598	OK303460	OK303519	OK303580	OK303647	Lin et al. 2024
* Cytospora parapersoonii*	T28.1 ^T^	AF191181	NA	NA	NA	NA	Adams et al. 2002
* Cytospora paraplurivora*	FDS-564 ^T^	OL640183	OL631587	NA	OL631590	NA	Ilyukhin et al. 2023
* Cytospora personata*	CBS 109774	PP988940	PQ074767	PQ075086	PQ074460	PQ075400	Lin et al. 2024
* Cytospora plurivora*	CBS 144239 ^T^	MG971861	MG972010	NA	MG971572	NA	Lawrence et al. 2018
* Cytospora polyspora*	CFCC 55834 ^T^	PP988944	PQ074770	PQ075089	PQ074463	PQ075403	Lin et al. 2024
* Cytospora polyspora*	CFCC 56012 ^T^	PP988945	PQ074771	PQ075090	PQ074464	PQ075404	Lin et al. 2024
* Cytospora polyspora*	CFCC 56014	PP988946	PQ074772	PQ075091	PQ074465	PQ075405	Lin et al. 2024
* Cytospora populi*	CFCC 55472 ^T^	MZ702609	OK303471	OK303530	OK303591	OK303658	Lin et al. 2023
* Cytospora populi*	CFCC 55473 ^T^	MZ702610	OK303472	OK303531	OK303592	OK303659	Lin et al. 2023
* Cytospora qinanensis*	JZB3670107 ^T^	PQ013669	NA	PQ053484	NA	PQ053504	He et al. 2024
* Cytospora regularis*	CFCC 71084 ^T^	PQ778595	PV454796	PV461982	NA	NA	Jiang et al. 2025a
* Cytospora regularis*	CFCC 71419 ^T^	PQ778596	PV454797	PV461983	NA	NA	Jiang et al. 2025a
* Cytospora rusanovii*	MFLUCC 15-0854 ^T^	KY417744	KY417710	KY417812	NA	NA	Norphanphoun et al. 2017
* Cytospora salicicola*	MFLUCC 14-1052 ^T^	KU982636	KU982637	NA	NA	NA	Li et al. 2016
* Cytospora salicis*	CBS 109754	PP988982	PQ074805	PQ075123	PQ074498	PQ075436	Lin et al. 2024
* Cytospora salicis-albae*	MFLUCC 18-0485 ^T^	MT734820	OL754585	OL754584	NA	NA	Manawasinghe et al. 2022
* Cytospora shangrilaensis*	CFCC 58247 ^T^	PP988989	PQ074812	PQ075130	PQ074504	PQ075443	Lin et al. 2024
* Cytospora shangrilaensis*	CFCC 58248 ^T^	PP988990	PQ074813	PQ075131	PQ074505	PQ075444	Lin et al. 2024
* Cytospora shannanensis*	CFCC 71118 ^T^	PQ778605	PV454806	PV461992	PV467218	NA	Jiang et al. 2025a
* Cytospora shannanensis*	CFCC 71300 ^T^	PQ778606	PV454807	PV461993	PV467219	PV467342	Jiang et al. 2025a
* Cytospora shawanensis*	CGMCC 3.18996 ^T^	PP965507	PP957865	PP957872	PP957879	PP957886	Cai et al. 2024
* Cytospora silvicola*	CFCC 71022 ^T^	PQ778609	PV454809	PV461996	PV467221	PV467343	Jiang et al. 2025a
*** Cytospora sinopopuli* sp. nov**.	**CFCC 71370** ^T^	**PX974050**	**PX981930**	**PX981934**	**PX981938**	**NA**	**This study**
*** Cytospora sinopopuli* sp. nov**.	**CFCC 71955** ^T^	**PX974051**	**PX981931**	**PX981935**	**PX981939**	**NA**	**This study**
* Cytospora sorbi*	MFLUCC 16-0631 ^T^	KY417752	KY417718	KY417820	NA	NA	Norphanphoun et al. 2017
* Cytospora sorbicola*	MFLUCC 16-0584 ^T^	KY417755	KY417721	KY417823	NA	NA	Norphanphoun et al. 2017
* Cytospora suecica*	CBS 450.51 ^T^	PP989015	PQ074834	PQ075156	PQ074530	PQ075467	Lin et al. 2024
* Cytospora tongzhouensis*	CFCC 56779 ^T^	PP989030	PQ074849	PQ075170	NA	PQ075480	Lin et al. 2024
* Cytospora translucens*	CFCC 58256	PP989033	PQ074852	PQ075172	PQ074544	PQ075482	Lin et al. 2024
* Cytospora xantha*	CFCC 71184 ^T^	PQ778623	PV454823	PV462007	PV467233	PV467356	Jiang et al. 2025a
* Cytospora xantha*	CFCC 71289 ^T^	PQ778624	PV454824	PV462008	NA	PV467357	Jiang et al. 2025a
* Cytospora yulinensis*	CFCC 89641 ^T^	KF765683	KU711006	KU710967	KU710929	KR045679	Fan et al. 2015b
* Cytospora yulinensis*	CFCC 71111	PQ778632	PV454831	PV462015	PV467241	PV467363	Jiang et al. 2025a

**Note**. “NA” indicates unavailable sequences; sequences produced in the current study are in bold, and “^T^” means ex-type strains.

Phylogenetic analyses were performed using maximum likelihood (ML) and Bayesian inference (BI) methods based on the concatenated five-gene dataset. ML analysis was conducted using IQ-TREE (Nguyen et al. 2015). The best-fit nucleotide substitution models for each gene partition were selected by ModelFinder (Kalyaanamoorthy et al. 2017) implemented in IQ-TREE, according to the Bayesian information criterion (BIC). Nodal support was assessed using 1,000 ultrafast bootstrap replicates (Hoang et al. 2018). Bayesian inference (BI) was performed using MrBayes v.3.2.6 (Ronquist et al. 2012). Two independent Markov chain Monte Carlo (MCMC) chains were run for 5,000,000 generations, with trees sampled every 1,000^th^ generation. The initial 25% of sampled trees were discarded as burn-in, and the remaining trees were used to calculate the consensus tree and posterior probabilities (PP). The resulting phylograms were visualized in FigTree v.1.4.2 and finalized using Adobe Illustrator (Rambaut 2014).

To evaluate the potential for genetic recombination and support species recognition, the pairwise homoplasy index (PHI) test was applied using SplitsTree v.4.16.1 (Huson and Bryant 2006). A PHI index (Фw) > 0.05 indicates a lack of significant recombination. Additionally, a split network based on the concatenated dataset was constructed using the NeighborNet algorithm to visualize potential conflicting signals.

## Results

### Phylogenetic analyses

***Cytospora kantschavelii* species complex**

**Figure 1.**
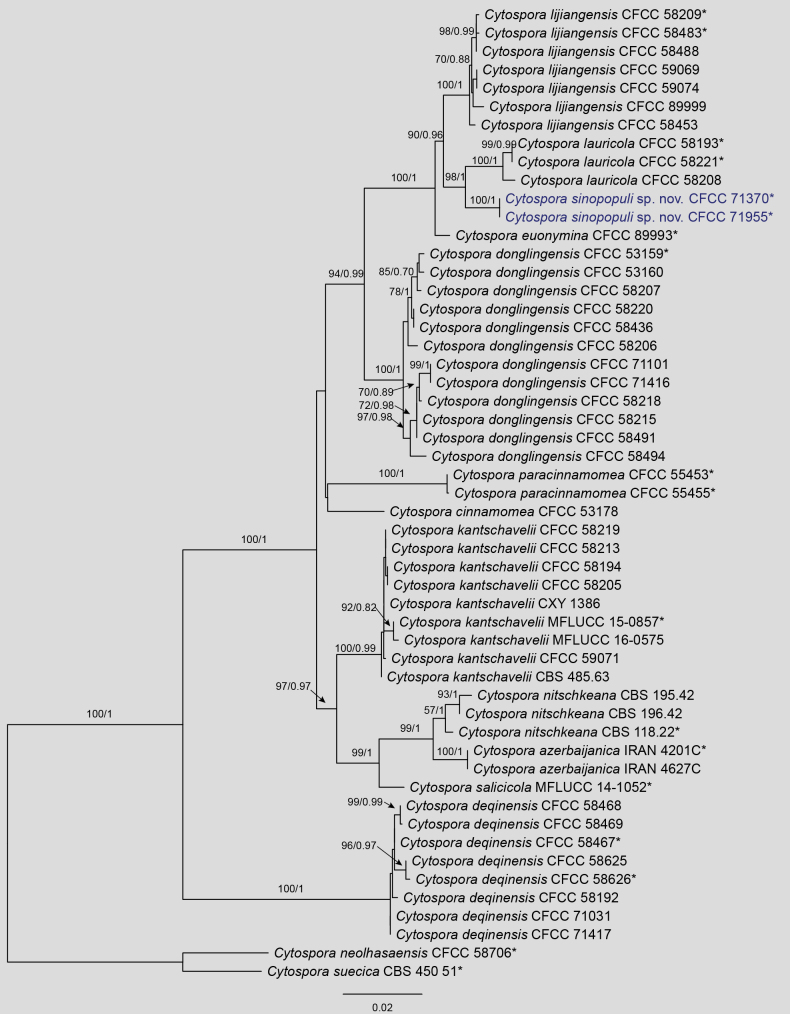
Maximum likelihood tree of the *Cytospora kantschavelii* species complex generated from combined ITS, *act*, *rpb*2, *tef*1, and *tub*2 sequence data. Bootstrap support values ≥ 50% and Bayesian posterior probabilities ≥ 0.90 are demonstrated at the branches. Isolates from the present study are indicated in blue, and * indicates ex-type strains.

The concatenated five-gene dataset (ITS, *act*, *rpb*2, *tef*1, and *tub*2) used to resolve the phylogeny of the *Cytospora kantschavelii* species complex included 53 strains belonging to 14 species. *Cytospora neolhasaensis* (CFCC 58706) and *C. suecica* (CBS 450.51) were utilized as the outgroup taxa. The final alignment consisted of 2,561 characters (including gaps), with the following partition lengths: *act* (1–252 bp), ITS (253–764 bp), *rpb*2 (765–1,634 bp), *tef*1 (1,635–2,119 bp), and *tub*2 (2,120–2,561 bp). The best-scoring ML tree (Fig. 1) resulted in a final likelihood value of −8351.53. According to the Bayesian information criterion (BIC), ModelFinder selected the following best-fit models: TNe+G4 for *act*, K2P+I for ITS, TNe+G4 for *rpb*2, TNe+G4 for *tef*1, and HKY+F+G4 for *tub*2. Phylogenetic inferences using both ML and BI yielded nearly identical topologies. The two new isolates from this study, CFCC 71370 and CFCC 71955, formed a distinct, monophyletic lineage with maximum statistical support (MLBS = 100%, BIPP = 1.0). This lineage, described here as *Cytospora sinopopuli*, is resolved as a sister group to *C. lauricola* (CFCC 58193, CFCC 58221, and CFCC 58208) with strong support (MLBS = 98%, BIPP = 1.0).

### *Cytospora leucostoma* species complex

**Figure 2.**
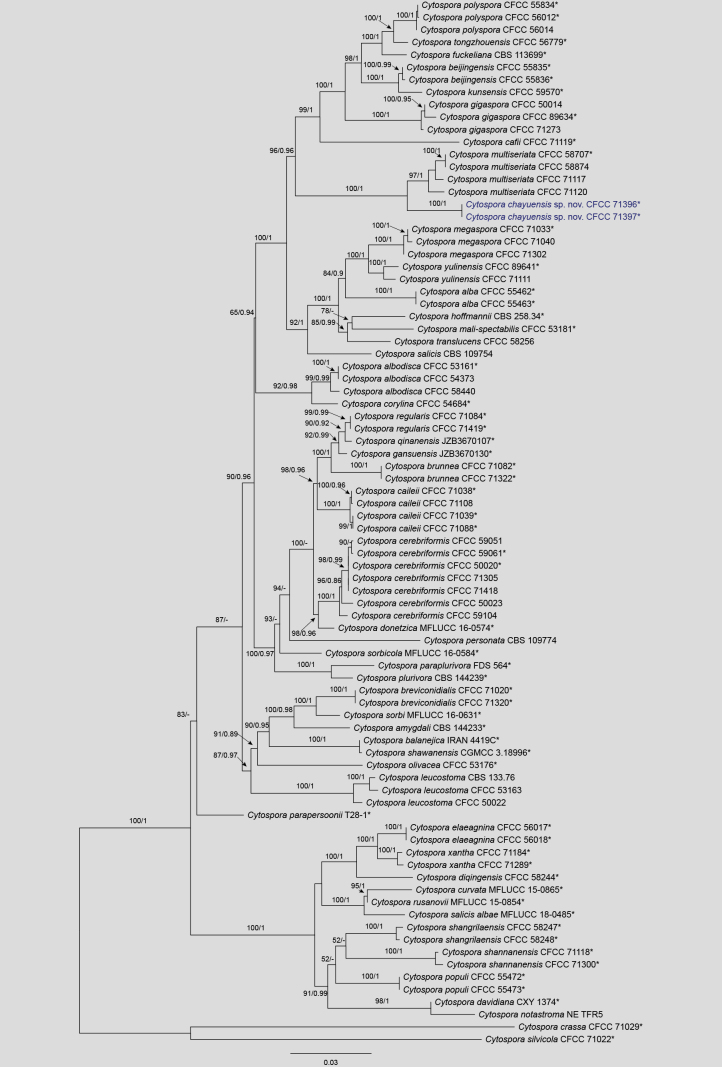
Maximum likelihood tree of the *Cytospora leucostoma* species complex generated from combined ITS, *act*, *rpb*2, *tef*1, and *tub*2 sequence data. Bootstrap support values ≥ 50% and Bayesian posterior probabilities ≥ 0.90 are demonstrated at the branches. Isolates from the present study are indicated in blue, and * indicates ex-type strains.

The concatenated five-gene dataset (ITS, *act*, *rpb*2, *tef*1, and *tub*2) used for the phylogenetic analysis of the *Cytospora leucostoma* species complex comprised 84 strains belonging to 50 species. *Cytospora crassa* (CFCC 71029) and *C. silvicola* (CFCC 71022) were selected as outgroup taxa. The final alignment contained 2,509 characters (including gaps), with individual partition lengths as follows: *act* (1–235 bp), ITS (236–743 bp), *rpb*2 (744–1,457 bp), *tef*1 (1,458–2,062 bp), and *tub*2 (2,063–2,509 bp). The best-scoring ML tree (Fig. 2) yielded a final likelihood value of −18001.91. Based on the BIC, ModelFinder selected the following best-fit models: K2P+G4 for *act*, SYM+I+G4 for ITS, TIM3e+I+G4 for *rpb*2, TIM2e+I+G4 for *tef*1, and HKY+F+G4 for *tub*2. Phylogenetic inferences using both ML and BI produced nearly identical topologies. The two new isolates, CFCC 71396 and CFCC 71397, formed a distinct and fully supported monophyletic lineage (MLBS = 100%, BIPP = 1.0). This lineage, described here as *Cytospora chayuensis*, clustered as a sister group to *C. multiseriata* (CFCC 58707, CFCC 58874, CFCC 71117, and CFCC 71120) with maximum statistical support (MLBS = 100%, BIPP = 1.0).

### PHI test

**Figure 3.**
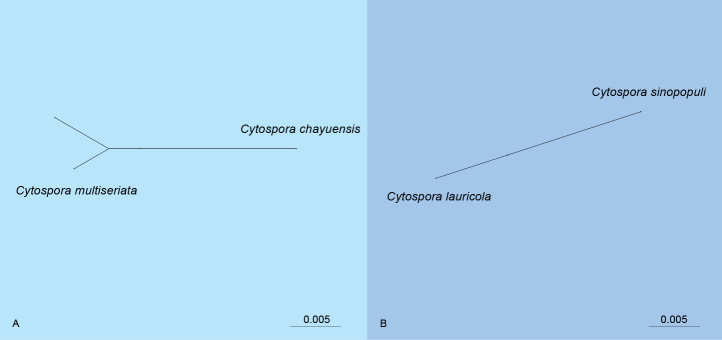
Phylogenetic network from concatenated data (ITS, *act*, *rpb*2, *tef*1, and *tub*2) representing the structure of *Cytospora* species, based on LogDet transformation and the NeighborNet algorithm, inferred by SplitsTree. The scale bar represents the expected number of substitutions per nucleotide position. **A**. *p* = 0.298; **B**. *p* = 1.0.

To assess the potential for genetic recombination between the newly proposed species and their closest phylogenetic relatives, a PHI analysis was conducted using the five-gene concatenated dataset (ITS, *act*, *rpb*2, *tef*1, and *tub*2). Two distinct clusters were selected for analysis based on the multi-locus phylogeny: Clade A included the new species *Cytospora chayuensis* and its closely related sister taxon, *C. multiseriata*. The PHI test resulted in a *p*-value of 0.298, indicating no significant evidence of genetic recombination between these lineages. Clade B comprised the new species *C. sinopopuli* and its sister taxon *C. lauricola*. This analysis yielded a *p*-value of 1.0, similarly suggesting an absence of significant recombination events within this group (Fig. 3).

### Taxonomy

#### Cytospora chayuensis

Taxon classificationFungiDiaporthalesValsaceae

Ning Jiang sp. nov.

5D368847-7799-50FE-9C3E-D01F935EA16E

862254

Fig. 4

##### Etymology.

Named after the collection site of the type specimen, Chayu County.

##### Description.

Associated with branch canker disease of *Salix lasiogyne*. **Teleomorph**: Undetermined. **Anamorph: *Conidiomata*** Group AII (type a7), pycnidial, scattered, immersed to semi-immersed in the bark, conical, 650–1000 μm diam., 350–600 μm high, with multiple subdivided locules with common walls. ***Conceptacle*** conspicuous. ***Ectostromatic disc*** white, circular to ovoid, 240–380 μm diam., with a single ostiole per disc in the center. ***Ostiole*** grey to brown, 75–140 μm diam. ***Conidiophores*** borne along the locules, hyaline, unbranched or branched at the base, (11.5–)13.5–24.5(–35) × (1–)1.5–2(–2.5) μm. ***Conidiogenous cells*** enteroblastic, phialidic, subcylindrical to cylindrical. ***Conidia*** hyaline, allantoid, thin-walled, aseptate, smooth, (7.5–)8.5–10.5(–11.5) × 1.5–2(–2.5) (av. = 9.5 ± 1 × 2.1 ± 0.2, *n* = 50) μm, L/W ratio = 4.5–5.2.

**Figure 4.**
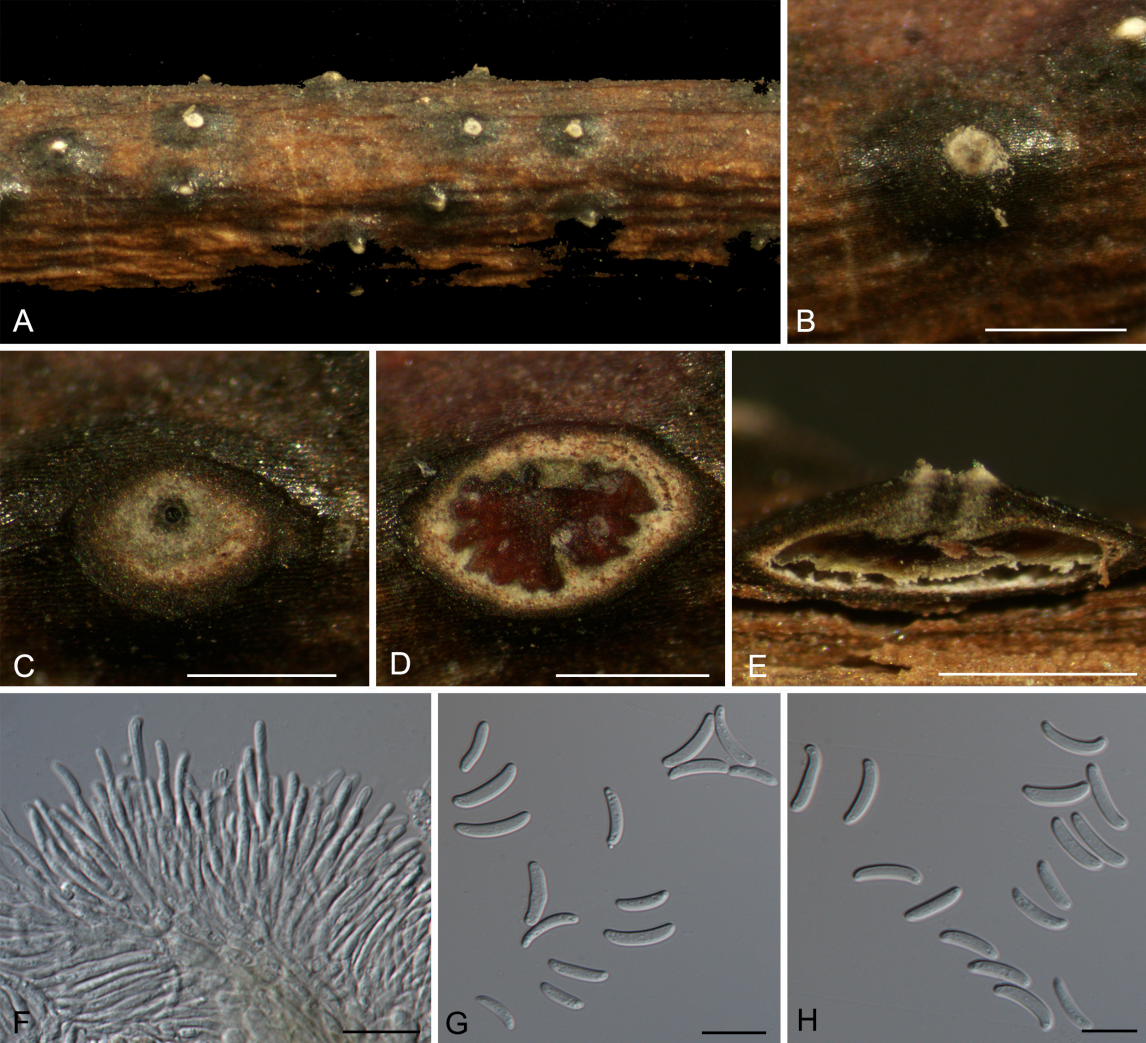
Morphology of *Cytospora chayuensis* on *Salix lasiogyne*. **A**. Habit of conidiomata on branch; **B**. Ectostromatic disc with ostiole; **C**. Ostiole in transverse section; **D**. Transverse section through conidioma; **E**. Conidiogenous cells with attached conidia; **F, G**. Conidia. Scale bars: 500 µm (**B–E**); 10 µm (**F–H**).

##### Culture characteristics.

Colonies on PDA flat, spreading, with moderate aerial mycelium and entire margin, smoke grey, fast growing, reaching 90 mm diam. after 1 wk at 25 °C, sterile.

##### Materials examined.

China, • Xizang Autonomous Region (Tibet), Linzhi (Nyingchi) City, Chayu (Zayü) County, 29°19'47"N, 97°6'3"E, 2192 m asl, from cankered branches of *Salix lasiogyne*, 22 Oct. 2024, *Ning Jiang, Min Liu, Jieting Li & Yi Li* (**holotype** CAF800150, ex-type cultures CFCC 71396 and CFCC 71397).

##### Notes.

*Cytospora chayuensis* sp. nov. is phylogenetically closely related to *C. multiseriata* (Fig. 2). Morphologically, *C. chayuensis* differs from *C. multiseriata* by possessing a conspicuous conceptacle and larger conidia (8.5–10.5 × 1.5–2 μm in *C. chayuensis* vs. 4–4.5 × 1.5 μm in *C. multiseriata*). Additionally, it differs from *C. multiseriata* by 5/513 bp (0.97%) in ITS, 2/238 bp (0.84%) in *act*, 4/726 bp (0.55%) in *rpb*2, 46/306 bp (15.03%) in *tef*1, and 29/401 bp (7.23%) in *tub*2 (Lin et al. 2024; Jiang et al. 2025a).

#### Cytospora sinopopuli

Taxon classificationFungiDiaporthalesValsaceae

Ning Jiang sp. nov.

94766CFB-3CBF-5DE4-A6AF-5908F33D26BB

862255

Fig. 5

##### Etymology.

Named after the collection country China (*sino*-) and the host genus, *Populus*.

##### Description.

Associated with branch canker disease of *Populus szechuanica* var. *tibetica*. **Teleomorph: *Pseudostromata*** Group SII (type s3), immersed in the bark, scattered to serried, conical, 1700–2450 μm diam., 450–800 μm high, with 6–12 perithecia arranged circularly. ***Conceptacle*** absent. ***Ectostromatic disc*** buff to honey, circular to ovoid, 420–600 μm diam., with 6–12 ostioles arranged circularly per disc. ***Ostioles*** brown to black, 85–150 μm diam. ***Perithecia*** flask-shaped to spherical, 350–500 μm diam. ***Asci*** hyaline, with chitinoid, refractive ring, clavate, (38.5–)45–59(–60) × (6.5–)9–13.5(–14.5) μm, 8-spored. ***Ascospores*** multiseriate, allantoid, thin-walled, hyaline, aseptate, (10–)13.5–15.5(–16.5) × (2–)2.5–3(–3.5) (av. = 14.6 ± 1.1 × 2.8 ± 0.3, *n* = 50) μm, L/W ratio = 4.7–5.7. **Anamorph**: Undetermined.

**Figure 5.**
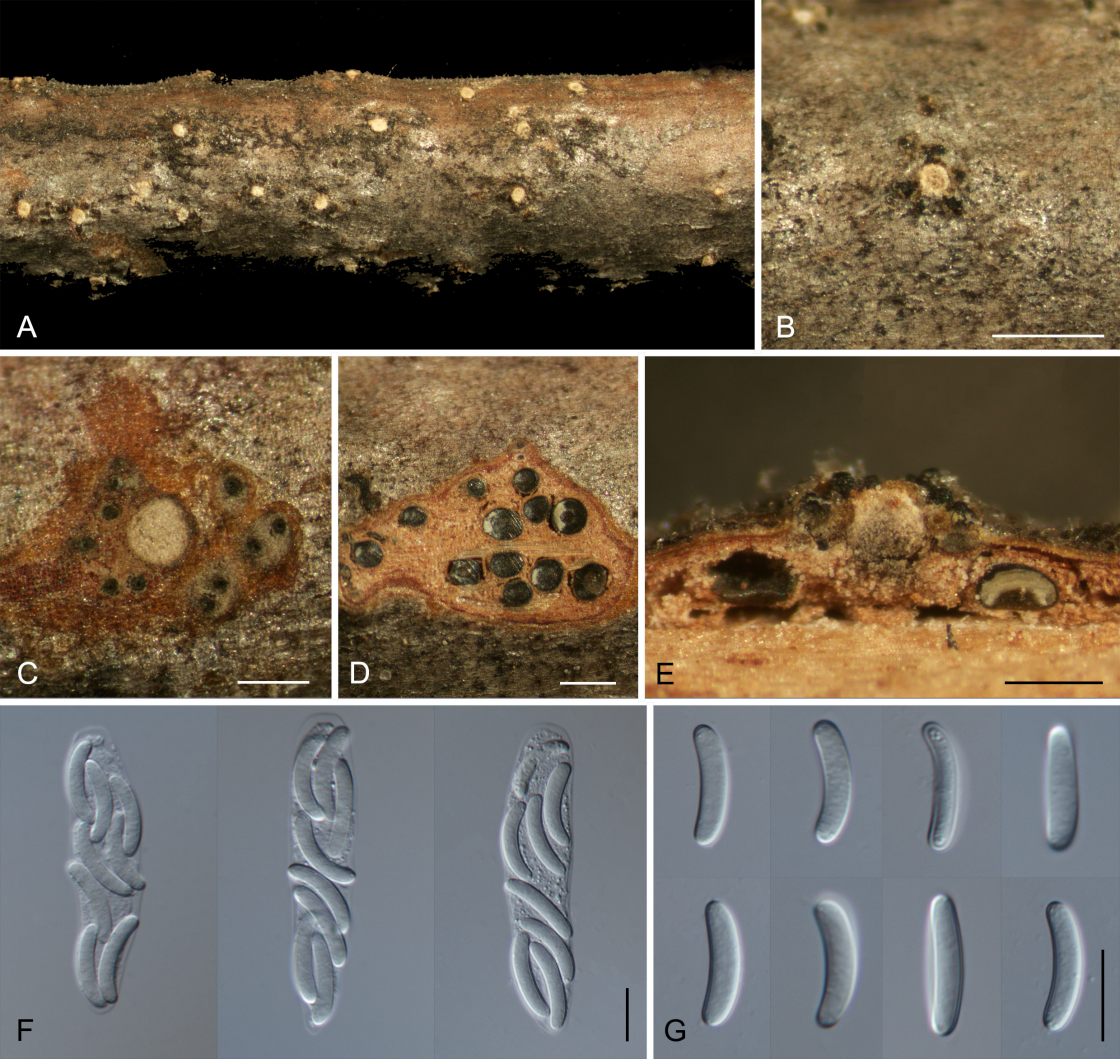
Morphology of *Cytospora sinopopuli* on *Populus szechuanica* var. *tibetica*. **A**. Habit of ascostromata on branch; **B**. Ectostromatic disc; **C**. Ostioles and pseudostroma in transverse section; **D**. Transverse section through ascostroma; **E**. Longitudinal section through ascostroma; **F**. Asci; **G**. Ascospores. Scale bars: 500 µm (**B–E**); 10 µm (**F, G**).

##### Culture characteristics.

Colonies on PDA flat, spreading, with abundant aerial mycelium and entire margin, initially white, turning brown after 1 wk, fast growing, reaching 90 mm diam. after 2 wk at 25 °C, sterile.

##### Materials examined.

China, • Xizang Autonomous Region (Tibet), Linzhi (Nyingchi) City, Bomi (Bomê) County, 29°44'1"N, 96°2'41"E, 3050 m asl, from cankered branches of *Populus szechuanica* var. *tibetica*, 22 Oct. 2024, *Ning Jiang, Min Liu, Jieting Li & Yi Li* (**holotype** CAF800151, ex-type cultures CFCC 71370 and CFCC 71955).

##### Notes.

*Cytospora sinopopuli*, isolated from *Populus szechuanica* var. *tibetica* (*Salicaceae*) in Xizang, is phylogenetically closely related to *C. lauricola* from *Laurus nobilis* (*Lauraceae*) in Yunnan (Fig. 1). Currently, *C. sinopopuli* is known only from its teleomorph, while *C. lauricola* has been described only in its anamorph stage; therefore, direct morphological comparison between the two species is not possible. Nevertheless, *C. sinopopuli* is clearly distinguished from *C. lauricola* by molecular data: 1/512 bp (0.19%) in ITS, 8/255 bp (3.14%) in *act*, 1/625 bp (0.16%) in *rpb*2, and 31/264 bp (11.74%) in *tef*1 (Lin et al. 2024).

## Discussion

The Qinghai-Xizang Plateau, often referred to as the “Third Pole,” represents a unique evolutionary arena characterized by extreme altitudes and diverse vertical climatic zones. Despite its ecological significance, the diversity of fungal pathogens in this region remains largely underexplored (Li et al. 2024b; Jiang et al. 2025a). In this study, the *Cytospora* species associated with canker diseases of *Salicaceae* in Xizang, China, were investigated. By integrating morphological characteristics with multi-locus phylogenetic analyses (ITS, *act*, *rpb*2, *tef*1, and *tub*2), two novel species, *Cytospora chayuensis* and *C. sinopopuli*, were identified.

The taxonomy of *Cytospora* has undergone a profound shift from a dual nomenclature system based on morphology and host specificity to a unified, polyphasic approach (Norphanphoun et al. 2017, 2018; Pan et al. 2018, 2020; Gao et al. 2021). Historically, species identification relied heavily on variable traits such as stromata size and the assumption of strict host specificity, which led to significant taxonomic confusion (Ehrenberg 1818; Wehmeyer 1975; Spielman 1985; Adams et al. 2002). The results reinforce the modern consensus that the “One Fungus, One Name” principle and multi-locus phylogeny are essential for accurate delimitation within this genus (Lin et al. 2024; Zhao et al. 2024; Jiang et al. 2025b; Wang et al. 2025). While the ITS region remains a primary barcode, it often lacks sufficient resolution for *Cytospora* species; thus, the inclusion of secondary markers such as *tef*1 and *tub*2 was critical in distinguishing the new species from their close relatives (Lin et al. 2024). No fixed sequence divergence threshold was applied for species delimitation in this study. Instead, the GCPSR framework, which relies on concordant phylogenetic placement across multiple independent gene trees (Suppl. material 1: figs S1–S10), was followed. The new species were consistently resolved as distinct lineages in single-gene phylogenies (particularly *rpb*2, *tef*1, and *tub*2) with no topological conflicts. While the observed divergence levels are generally consistent with those reported in recent *Cytospora* studies, species delimitation was based on an integrative approach combining phylogenetic concordance, morphological characters, and host association, rather than any arbitrary cutoff (Lin et al. 2024; Jiang et al. 2025a).

*Cytospora chayuensis* is assigned to the *Cytospora leucostoma* species complex, a group that exhibits host specificity toward *Rosaceae* and *Salicaceae* plant families (Jiang et al. 2025a). Phylogenetically, *C. chayuensis* forms a fully supported sister clade to *C. multiseriata*. However, several lines of evidence support their separation as distinct biological entities. Morphologically, *C. chayuensis* possesses a conspicuous conceptacle and significantly larger conidia (8.5–10.5 × 1.5–2 μm) compared to those of *C. multiseriata* (4–4.5 × 1.5 μm) (Lin et al. 2024; Jiang et al. 2025a). This morphological divergence is mirrored by significant sequence variation at the molecular level, most notably in the *tef*1 (15.03%) and *tub*2 (7.23%) loci, which far exceeds the typical interspecific threshold for the genus. Furthermore, the PHI test result (*p* = 0.298) indicates an absence of significant recombination.

*Cytospora sinopopuli* resides within the *Cytospora kantschavelii* species complex and is phylogenetically related to *C. lauricola*. While *C. sinopopuli* was described from its teleomorph on *Populus szechuanica* var. *tibetica*, its sister species *C. lauricola* is currently known only from its anamorph on *Laurus nobilis* (Lin et al. 2024). This difference in known life stages makes direct morphological comparison difficult; however, the molecular data are definitive. The two species differ by 31 base pairs (11.74%) in the *tef*1 region, and the PHI test (*p* = 1.0) indicates an absence of significant recombination. The discovery of *C. sinopopuli* on a *Salicaceae* host at high altitude aligns with recent findings that certain *Cytospora* lineages exhibit tight host associations or are driven by specialized evolutionary pressures in extreme environments.

The discovery of *C. chayuensis* and *C. sinopopuli* in the high-altitude ecosystems of Xizang provides significant insights into the evolutionary radiation and host-association patterns within the genus. While many *Cytospora* species are known for their broad host ranges, the findings suggest that the extreme environmental pressures of the “Third Pole,” including intense UV radiation and drastic diurnal temperature fluctuations, may act as drivers for niche specialization and speciation (Jiang et al. 2025c). The significant genetic distance observed in the *tef*1 and *tub*2 loci between *C. chayuensis* and *C. multiseriata*, as well as between *C. sinopopuli* and *C. lauricola*, far exceeds the typical interspecific thresholds reported in recent revisions of the genus (Lin et al. 2024). This high level of divergence, coupled with the lack of genetic recombination confirmed by PHI tests, indicates that these taxa are not merely environmental variants but stable, independent evolutionary lineages. Furthermore, the presence of *C. sinopopuli* on *Populus* at 3,050 m asl, contrasted with its sister species *C. lauricola* found on *Laurus* in more temperate regions (Lin et al. 2024), underscores a potential host shift or range expansion driven by altitude-induced isolation. These findings challenge the traditional reliance on morphological stasis in *Cytospora* and highlight the necessity of sampling extreme environments to fully resolve the taxonomic boundaries and host-preference evolution within the *Cytosporaceae*. It should be noted that the present study focuses primarily on taxonomy rather than pathology. Koch’s postulates were not conducted to confirm the pathogenicity of the newly described species. Therefore, whether these fungi act as primary pathogens or secondary colonizers of stressed hosts remains to be determined. Future studies incorporating pathogenicity tests are needed to clarify their ecological roles.

Whether the new species described in this study are endemic to the Tibetan Plateau or more widely distributed across other high-altitude regions remains unclear due to limited sampling from these areas. Comparative studies incorporating broader geographic sampling are needed to test the hypothesis of endemic diversification in high-altitude ecosystems. Poplars and willows are vital pioneer species for agroforestry and ecosystem maintenance in the plateau environment. The identification of *C. chayuensis* and *C. sinopopuli* as fungi associated with canker diseases provides foundational data for forest health management in Xizang. These fungi often transition from latent endophytes to aggressive pathogens when hosts are stressed by the high-altitude conditions of the Tibetan Plateau, such as intense UV radiation, drought, or frost. This study not only enriches understanding of *Cytospora* diversity in high-altitude hotspots but also highlights the importance of surveys in understudied regions to uncover the true breadth of fungal life.

## Supplementary Material

XML Treatment for Cytospora chayuensis

XML Treatment for Cytospora sinopopuli

## Additional information

### Artificial Intelligence (AI) use

The authors accept full responsibility for the content of the manuscript, including the disclosure of any use of AI.

No AI tools were used in the preparation of this manuscript.

### Author ORCIDs

J. Li https://orcid.org/0000-0002-6679-5227

Y. Li https://orcid.org/0009-0004-0656-9799

Y. Wang https://orcid.org/0000-0003-0993-8102

N. Jiang https://orcid.org/0000-0002-9656-8500

### Data availability

All of the data that support the findings of this study are available in the main text or Supplementary Information.

## Supplementary materials

Supplementary material 1 Supplementary figures https://doi.org/10.3897/mycokeys.132.187445.suppl1 Data type docx Explanation note **fig. S1**. Maximum likelihood tree of *Cytospora kantschavelii* species complex generated from ITS sequence data. **fig. S2**. Maximum likelihood tree of *Cytospora kantschavelii* species complex generated from *act* sequence data. **fig. S3**. Maximum likelihood tree of *Cytospora kantschavelii* species complex generated from *rpb*2 sequence data. **fig. S4**. Maximum likelihood tree of *Cytospora kantschavelii* species complex generated from *tef*1 sequence data. **fig. S5**. Maximum likelihood tree of *Cytospora kantschavelii* species complex generated from *tub*2 sequence data. **fig. S6**. Maximum likelihood tree of *Cytospora leucostoma* species complex generated from ITS sequence data. **fig. S7**. Maximum likelihood tree of *Cytospora leucostoma* species complex generated from *act* sequence data. **fig. S8**. Maximum likelihood tree of *Cytospora leucostoma* species complex generated from *rpb*2 sequence data. **fig. S9**. Maximum likelihood tree of *Cytospora leucostoma* species complex generated from *tef*1 sequence data. **fig. S10**. Maximum likelihood tree of *Cytospora leucostoma* species complex generated from *tub*2 sequence data. https://binary.pensoft.net/file/1631067This dataset is made available under the Open Database License (http://opendatacommons.org/licenses/odbl/1.0/). The Open Database License (ODbL) is a license agreement intended to allow users to freely share, modify, and use this Dataset while maintaining this same freedom for others, provided that the original source and author(s) are credited. Jiangrong Li, Yi Li, Yonglin Wang, Ning Jiang
