# Supplementary material for: Identification of Cytospora (Cytosporaceae, Diaporthales) species associated with poplar and willow canker diseases in Xizang, China
Source: MycoKeys. 2026 May 8;132:41–61. doi: 10.3897/mycokeys.132.187445 (PMC13179503; doi:10.3897/mycokeys.132.187445)
Supplement: Supplementary material 1 — Supplementary figures [file mycokeys-132-041-s001.docx]

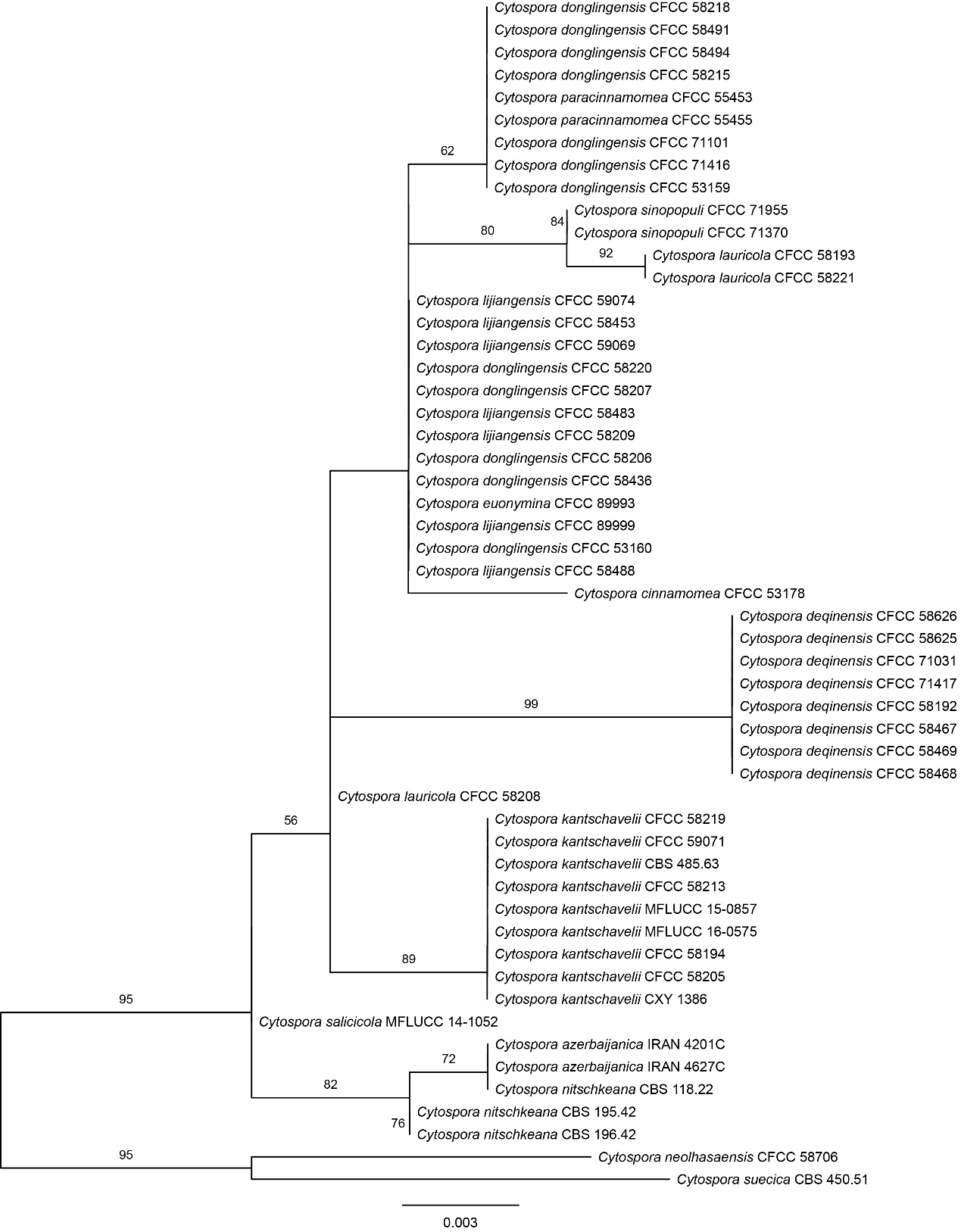


**Figure S1.** Maximum likelihood tree of *Cytospora kantschavelii* species complex generated from ITS sequence data. Bootstrap support values ≥ 50% are demonstrated at the branches.


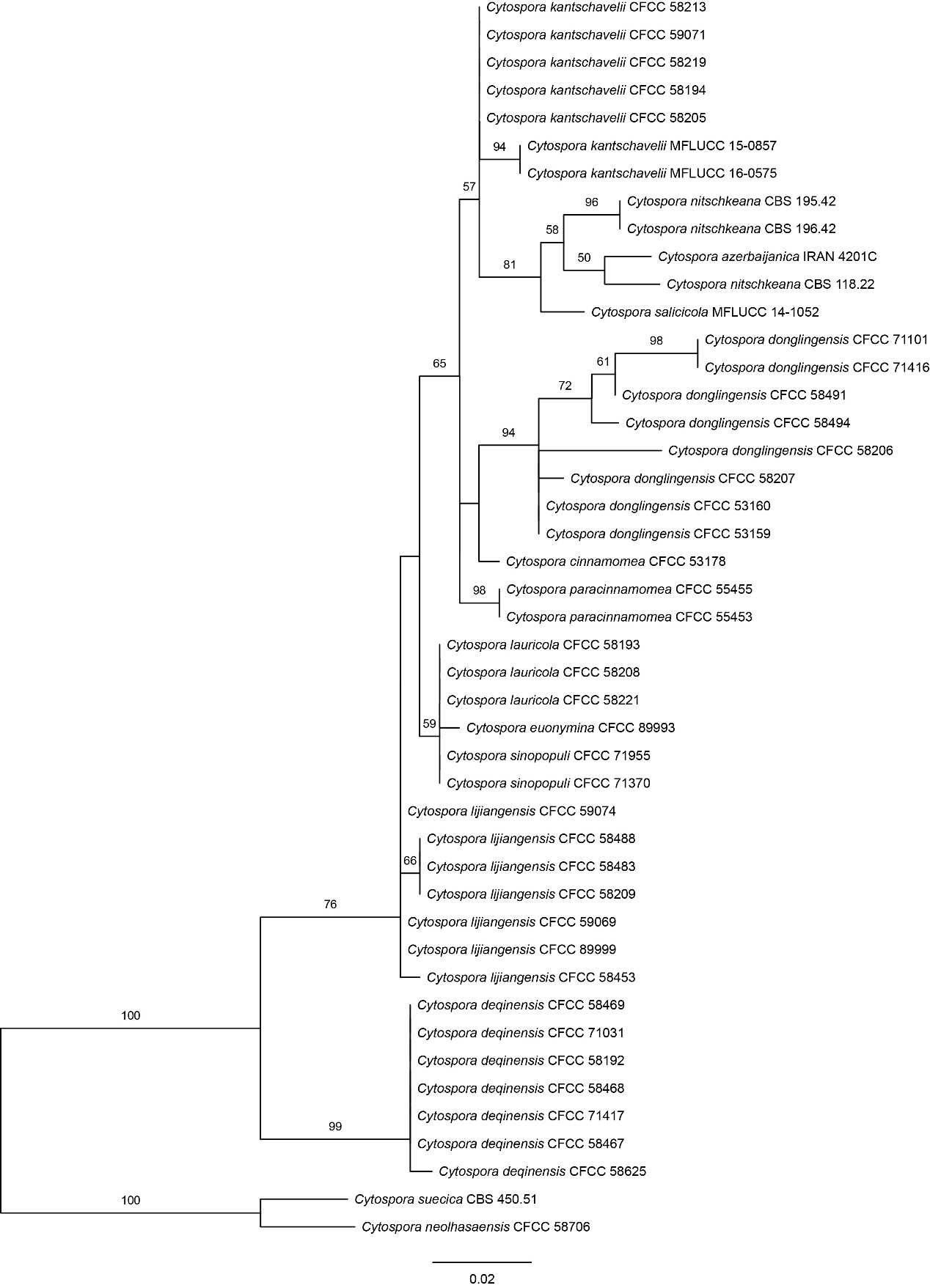


**Figure S2.** Maximum likelihood tree of *Cytospora kantschavelii* species complex generated from *act* sequence data. Bootstrap support values ≥ 50% are demonstrated at the branches.


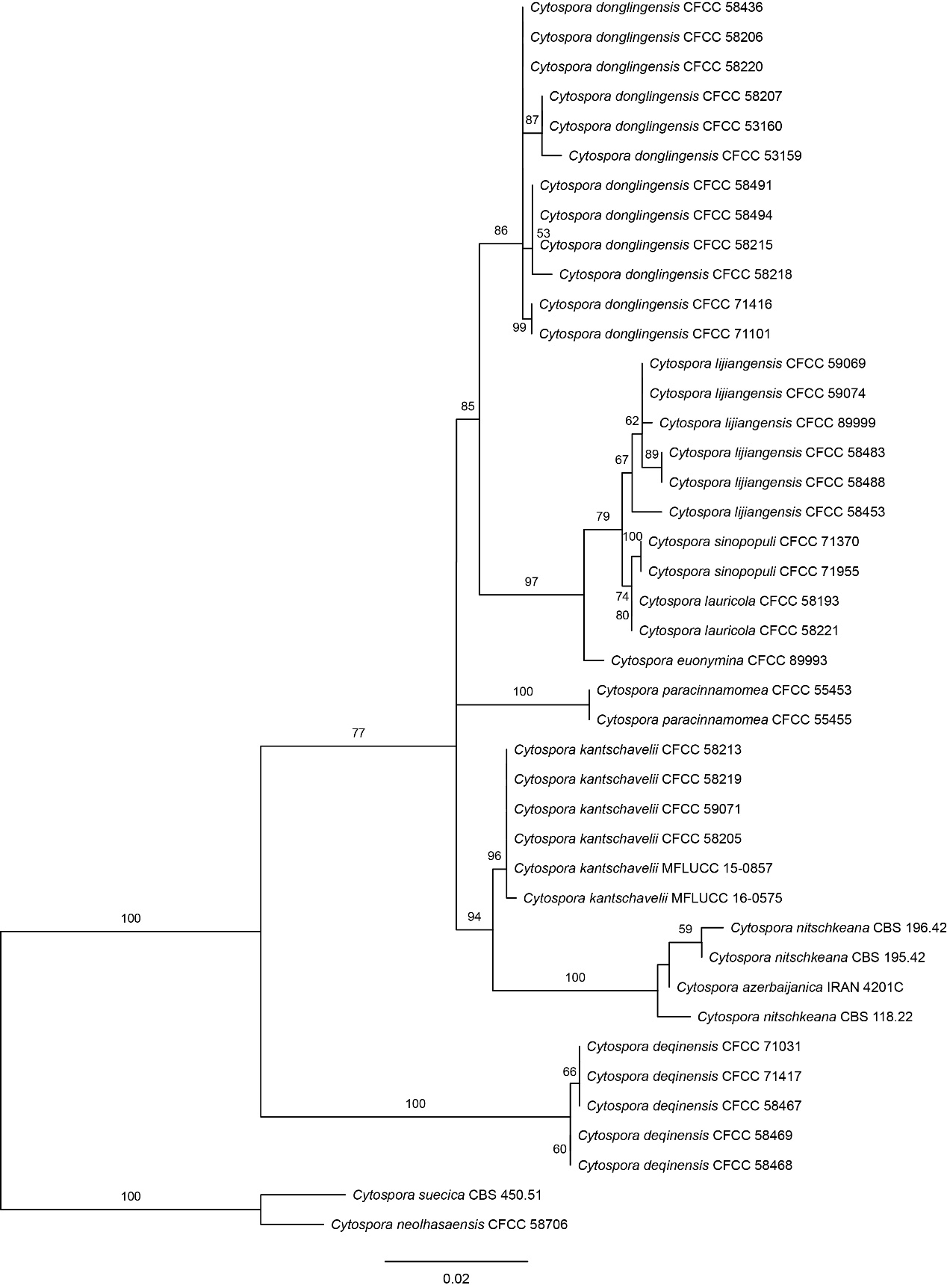


**Figure S3.** Maximum likelihood tree of *Cytospora kantschavelii* species complex generated from *rpb2* sequence data. Bootstrap support values ≥ 50% are demonstrated at the branches.


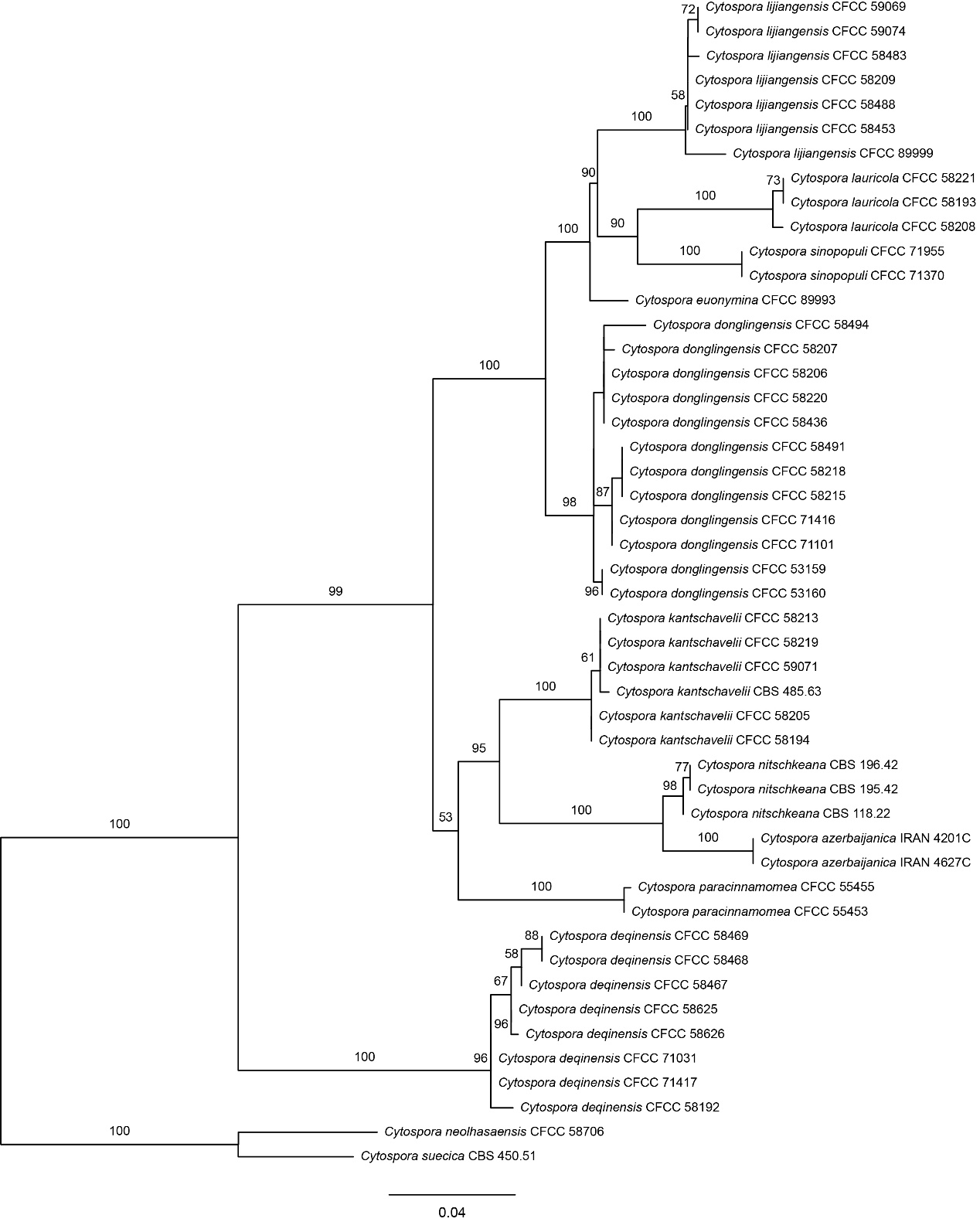


**Figure S4.** Maximum likelihood tree of *Cytospora kantschavelii* species complex generated from *tef1* sequence data. Bootstrap support values ≥ 50% are demonstrated at the branches.


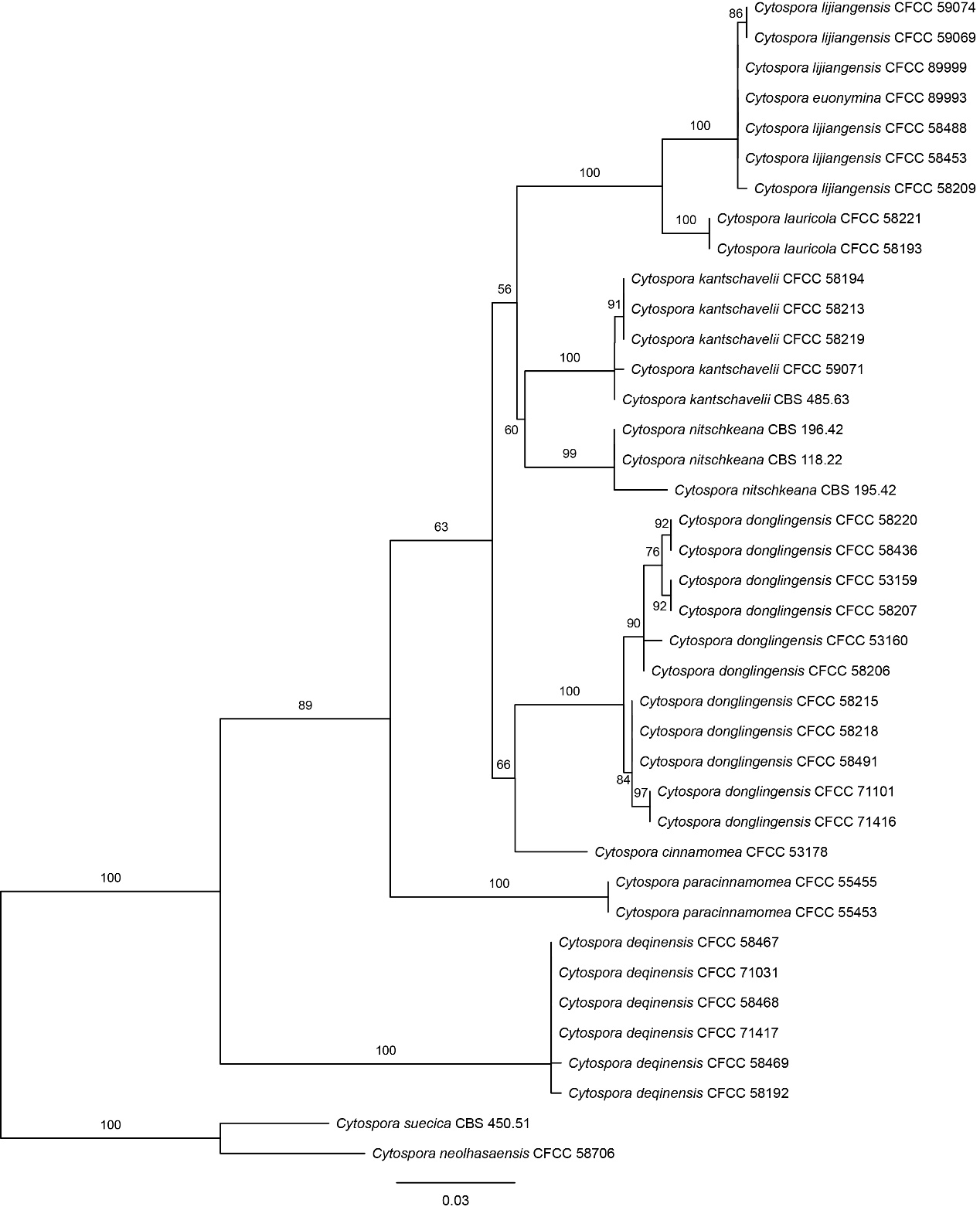


**Figure S5.** Maximum likelihood tree of *Cytospora kantschavelii* species complex generated from *tub2* sequence data. Bootstrap support values ≥ 50% are demonstrated at the branches.


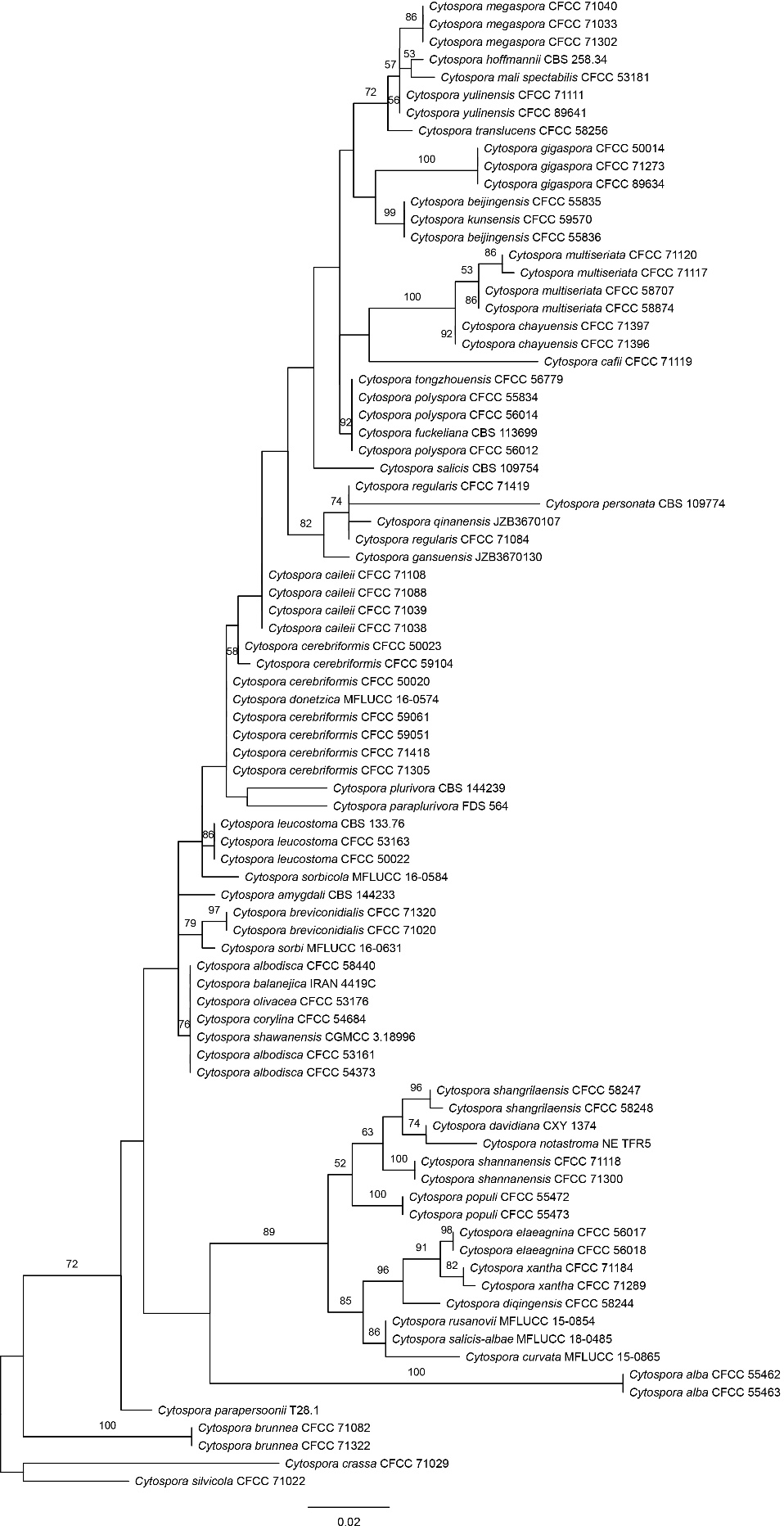


**Figure S6.** Maximum likelihood tree of *Cytospora leucostoma* species complex generated from ITS sequence data. Bootstrap support values ≥ 50% are demonstrated at the branches.


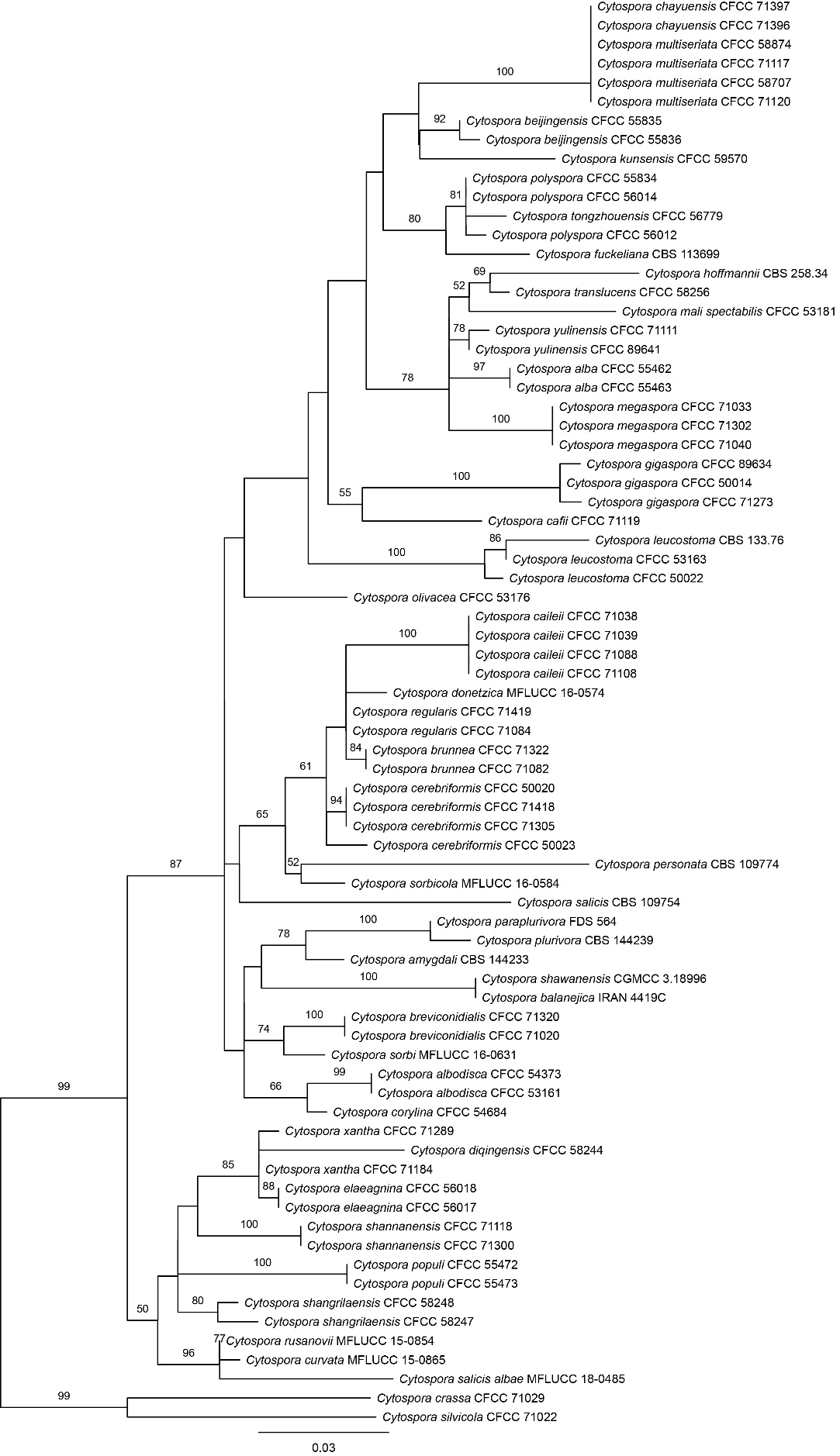


**Figure S7.** Maximum likelihood tree of *Cytospora leucostoma* species complex generated from *act* sequence data. Bootstrap support values ≥ 50% are demonstrated at the branches.


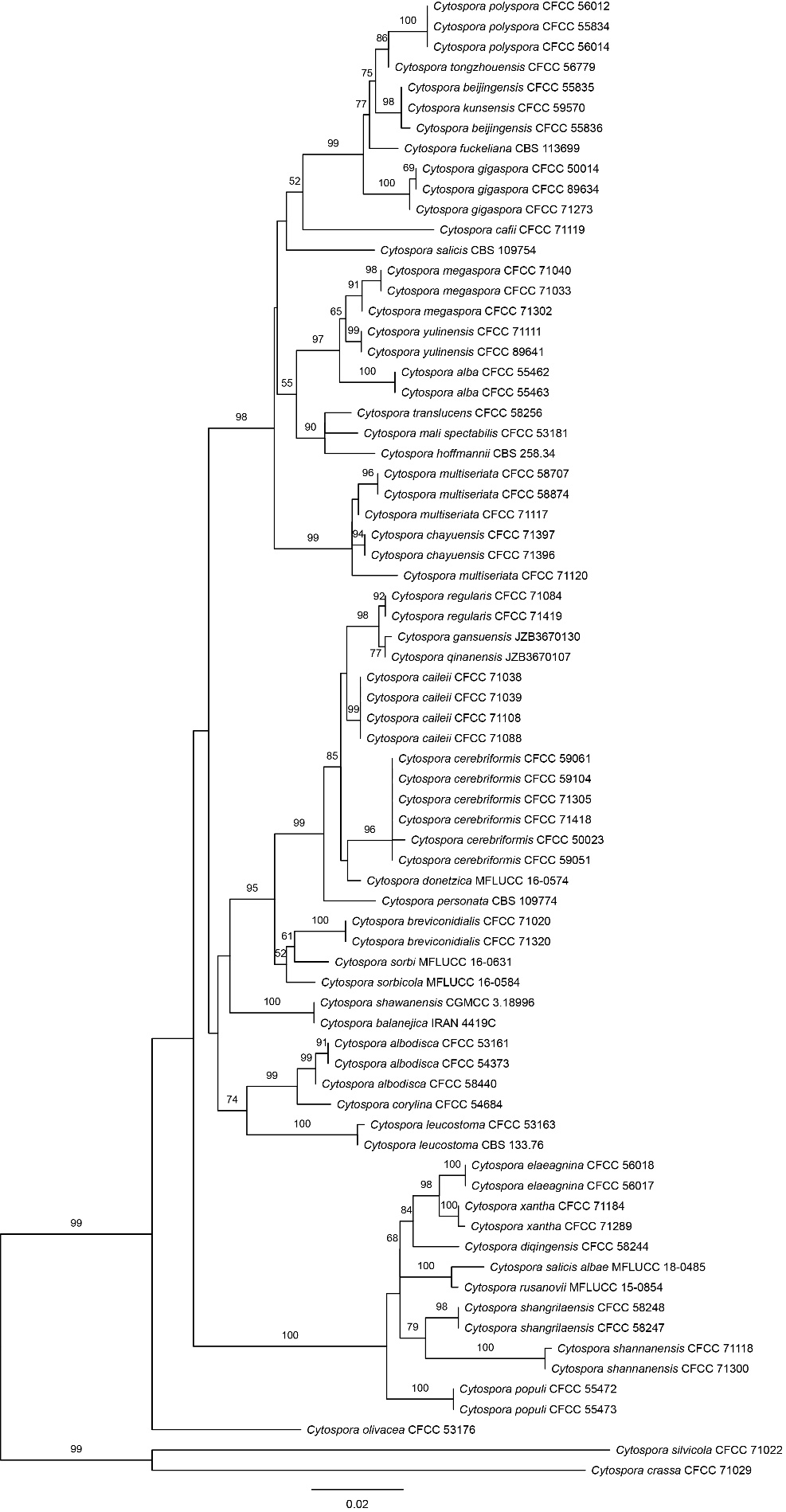


**Figure S8.** Maximum likelihood tree of *Cytospora leucostoma* species complex generated from *rpb2* sequence data. Bootstrap support values ≥ 50% are demonstrated at the branches.


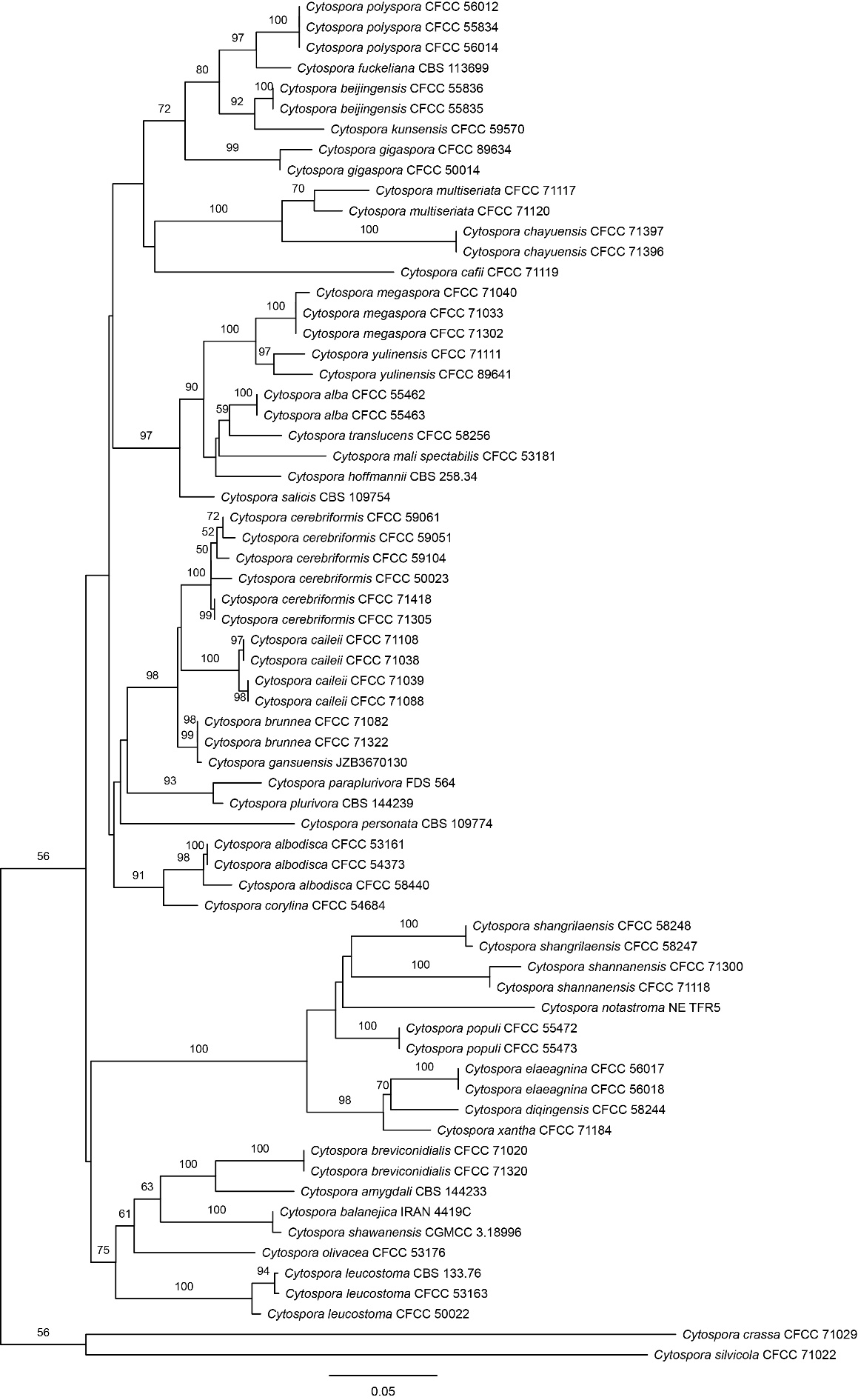


**Figure S9.** Maximum likelihood tree of *Cytospora leucostoma* species complex generated from *tef1* sequence data. Bootstrap support values ≥ 50% are demonstrated at the branches.


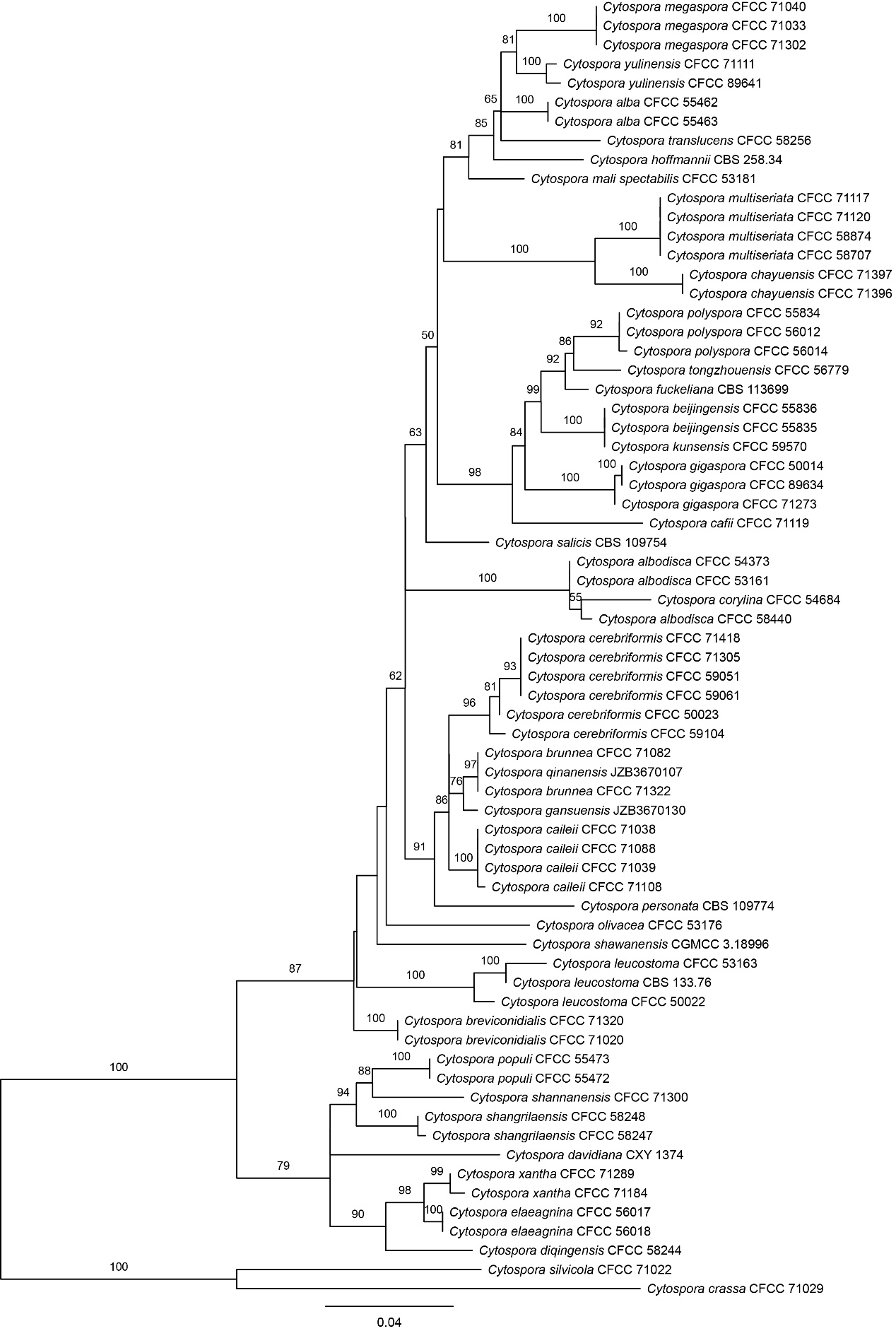


**Figure S10.** Maximum likelihood tree of *Cytospora leucostoma* species complex generated from *tub2* sequence data. Bootstrap support values ≥ 50% are demonstrated at the branches.
